# Ecosystem Carbon Stock Influenced by Plantation Practice: Implications for Planting Forests as a Measure of Climate Change Mitigation

**DOI:** 10.1371/journal.pone.0010867

**Published:** 2010-05-27

**Authors:** Chengzhang Liao, Yiqi Luo, Changming Fang, Bo Li

**Affiliations:** 1 Coastal Ecosystems Research Station of the Yangtze River Estuary, Ministry of Education Key Laboratory for Biodiversity Science and Ecological Engineering, Institute of Biodiversity Science, Fudan University, Shanghai, China; 2 Department of Botany and Microbiology, University of Oklahoma, Norman, Oklahoma, United States of America; University of Zurich, Switzerland

## Abstract

Uncertainties remain in the potential of forest plantations to sequestrate carbon (C). We synthesized 86 experimental studies with paired-site design, using a meta-analysis approach, to quantify the differences in ecosystem C pools between plantations and their corresponding adjacent primary and secondary forests (natural forests). Totaled ecosystem C stock in plant and soil pools was 284 Mg C ha^−1^ in natural forests and decreased by 28% in plantations. In comparison with natural forests, plantations decreased aboveground net primary production, litterfall, and rate of soil respiration by 11, 34, and 32%, respectively. Fine root biomass, soil C concentration, and soil microbial C concentration decreased respectively by 66, 32, and 29% in plantations relative to natural forests. Soil available N, P and K concentrations were lower by 22, 20 and 26%, respectively, in plantations than in natural forests. The general pattern of decreased ecosystem C pools did not change between two different groups in relation to various factors: stand age (<25 years vs. ≥25 years), stand types (broadleaved vs. coniferous and deciduous vs. evergreen), tree species origin (native vs. exotic) of plantations, land-use history (afforestation vs. reforestation) and site preparation for plantations (unburnt vs. burnt), and study regions (tropic vs. temperate). The pattern also held true across geographic regions. Our findings argued against the replacement of natural forests by the plantations as a measure of climate change mitigation.

## Introduction

Forest plantations (plantations) have been advocated as a measure to sequestrate carbon (C) from the atmosphere and to mitigate future climate change [1]. The global area of plantations was as large as 1.39×10^8^ ha in 2005, and the relative rate of annual expansion is predicted to be 2% approximately [2]. Reforestation in the lands where primary and secondary forests were harvested accounts for about half of total increased area of plantations [2]. Primary and secondary forests (a shorter term ‘natural forests’ used below) are considered as a large reservoir of C stock in terrestrial ecosystems [3], [4]. Whether or not plantations have the same ecosystem C stock as natural forests has drawn much attention [e.g., [3], [5], [6]]. Quantification of the difference in ecosystem C stock between them can directly come from field studies [e.g., [5]–[8]]. Although these studies are highly valued, the results are of high inconsistence, which precludes generalizing the roles of plantations in C stock on a global scale.

The inconsistent results may be associated with various factors including stand types and land-use history of plantations, and climatic and geographic conditions in study sites. Aboveground biomass is larger in plantations afforested in non-forested lands [9], but smaller in those reforested in natural forests than that in their corresponding adjacent natural forests [e.g., [5], [10]]. Aboveground litter mass is lower in plantations with an age of ten years [11], but higher in those with an age of 48 years than that in natural forests [12]. Belowground biomass is larger in plantations with evergreen coniferous species of *Picea abies* [13] and *Pinus ponderosa* [14], but smaller in those with deciduous broadleaved species of *Populus deltoids* than that in natural forests [15]. Soil C stock is lower in plantations in tropics [e.g., [9], [16], [17]], but higher than that in natural forests in temperate regions [e.g., [15], [18], [19]]. Additionally, origin (native or exotic) of tree species [e.g., [5], [16], [20]] and site preparation (unburnt or burnt treatment) [e.g., [5], [13], [14]] for plantation establishment may influence the difference in ecosystem C stock between plantations and natural forests. However, the individual field studies can not be used to explore the general patterns of such differences in relation to these factors.

The inconsistent results may stem from the fact that individual studies do often not provide much information on ecosystem processes, which is helpful for our understanding of why plantations differ in ecosystem C stock from natural forests. For example, lower aboveground net primary production (ANPP), aboveground litterfall, and fine root biomass lead to lower C sequestration into ecosystems [21]. Lower soil available nitrogen (N), phosphorus (P) and potassium (K) concentrations constrain tree growth and thus, limit ecosystem C sequestration. To understand the difference in ecosystem C stock, it is necessary to examine the differences in ecosystem C fluxes and relevant parameters, and soil nutrient availabilities.

Several syntheses have been conducted to explore the effects of plantations on ecosystem C stock, but they focused on the comparison of soil C stock between plantations and non-forested lands [e.g., [7], [8], [22]]. In this study, field studies with paired-site design were synthesized, using a meta-analysis approach, to search for a general pattern of the difference in ecosystem C stock between plantations and natural forests. Variables related to ecosystem C pools in above- and belowground biomass, aboveground litter mass, and soil C stock were included in this meta-analysis. Variables of ecosystem C fluxes including ANPP, aboveground litterfall and rate of soil respiration, and C parameters associated with fine root biomass, soil C concentration and soil microbial C concentration were analyzed. In addition, this synthesis examined the differences in soil available N, P and K concentrations. Specifically, the meta-analysis was performed to address the following three questions. First, to what extent ecosystem C stock was different between plantations and natural forests? Second, which factors contributed to the difference? Third, what were the consequences of plantation practice to global C cycle?

## Results

A total of 86 published studies with paired-site design were synthesized (References S1), in which arboreal species for plantations were included whereas shrubs, fruit and non-timber species such as apple, rubber and coffee trees were excluded from this analysis. The constructed database consisting of 373 lines of entries was used to compute the response ratios of variables (Table S1). The database covered 26 countries, but most studies were conducted in four countries: China, USA, Brazil, and Australia. The most common four species used for growing plantation forests were *Cunninghamia lanceolata*, *Pinus caribaea*, *P*. *radiata* and *Picea abies*, and consequently most of the plantations considered were pure coniferous stands. Mean age of plantations was 30 years with a range from 4 to 80 years (Table 1). Mean depth of soil samples for measured soil variables was 30 cm with a range from 5 to 120 cm (Table 1).

**Table 1:** Description of the variables in this analysis, with numbers of published papers and positive and negative cases in plantations relative to natural forests, mean and its range of plantation age and soil depth for this meta-analysis.

Variables		Number of				Plantation age (year)		Soil depth (cm)	
		Papers	Cases			Mean	Range	Mean	Range
			Total	Negative	Positive				
	Aboveground net primary production	4	9	6	3	43	32 to 50	-	-
	Aboveground litterfall	11	28	19	9	27	5 to 55	-	-
	Rate of soil respiration	8	14	12	2	33	12 to 60	-	-
	Aboveground biomass	11	20	17	3	26	9 to 55	-	-
	Aboveground litter mass	16	34	20	14	25	4 to 53	-	-
	Belowground biomass	8	17	13	4	21	5 to 55	72	0 to 30–120
	Soil C stock	25	51	41	10	27	9 to 75	33	0 to 5–100
	Fine root biomass	11	20	16	4	30	4 to 70	48	0 to 10–120
	Soil C concentration	50	84	68	15	31	7 to 80	17	0 to 5–100
	Soil microbial C concentration	12	19	17	2	27	5 to 75	16	0 to 10–30
	Soil available N concentration	9	25	20	5	38	9 to 72	18	0 to 5–40
	Soil available P concentration	14	32	19	12	32	9 to 73	29	0 to 10–100
	Soil available K concentration	12	20	15	5	24	9 to 50	17	0 to 5–20

**Figure 1:**
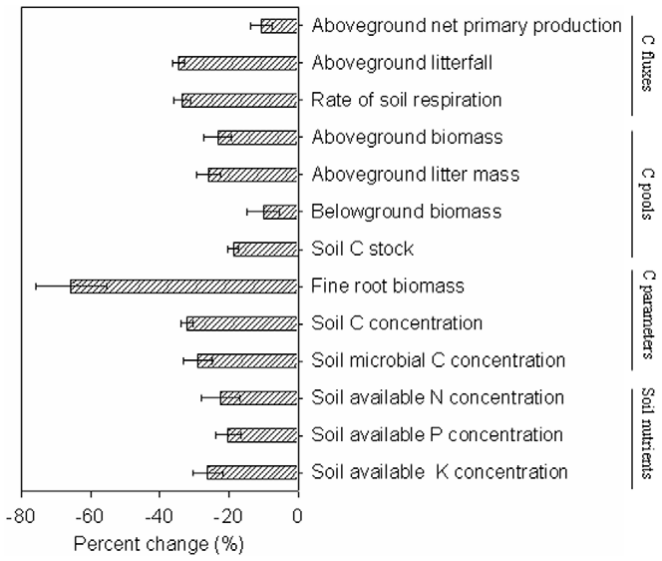
Percent changes of variables related to ecosystem C fluxes and pools, and soil nutrients in plantations relative to natural forests. Bars represented mean±95% confidence interval (CI).

Our meta-analysis showed that plantations had significantly lower ecosystem C pools including those in above- and belowground biomass, aboveground litter mass, and soil than natural forests (Fig. 1). Totaled ecosystem C stock was 205 and 284 Mg C ha^−1^ for plantations and natural forests, respectively (Table 2). ANPP, aboveground litterfall and rate of soil respiration were respectively 11, 34, and 32% lower, in plantations than in natural forests (Fig. 1). Fine root biomass, soil C concentration, and soil microbial C concentration decreased respectively by 66, 32, and 29% in plantations in comparison with natural forests (Fig. 1). Moreover, soil available N, P and K concentrations were respectively 22, 20 and 26% lower in plantations when compared with natural forests (Fig. 1).

**Table 2:** Ecosystem C pools (Mg C ha^−1^) in plantations and natural forests.

Component	Plantations	Natural forests
Aboveground biomass	79.5±11.9	121.2±14.9
Aboveground litter mass	5.1±0.6	6.1±0.8
Belowground biomass†	16.8±2.3	28.0±3.7
Soil C stock‡	103.9±10.1	128.8±13.7
Total	205.2	284.1

Note: Ecosystem C pools were given as mean±1SE.

†: Sampling depth up to a range from 0 to 30–120 cm where the large proportion of belowground biomass had been harvested [13].

‡: Soil C stock within the depth of 100 cm was calculated by a simple model: *Y* = *a* [1−exp (−b/x)] (see Materials and Methods).

**Figure 2:**
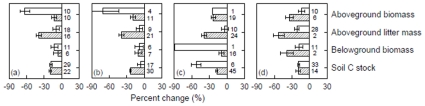
Percent change of ecosystem C pools from natural forests to plantations with two different groups in relation to stand age (a), stand type (b and c), and tree origin (d). Bars represented mean±95% CI. Values near each bar indicates the number of cases synthesized. Note: open bar- (a) <25 years, (b) broadleaved, (c) deciduous, and (d) native; hatched bar- (a) ≥25 years, (b) coniferous, (c) evergreen, and (d) exotic.

**Figure 3 pone-0010867-g003:**
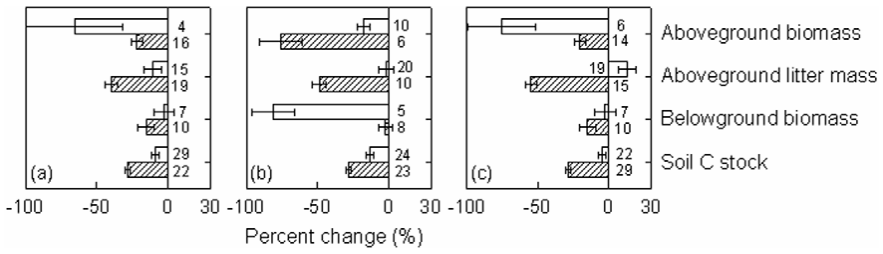
Percent change of ecosystem C pools from natural forests to plantations with two different groups in relation to land-use history (a), site preparation (b) for plantations, and study regions (c). Bars represented mean±95% CI. Values near each bar indicated the number of cases synthesized. Note: open bar- (a) afforestation, (b) unburnt, and (c) tropic; hatched bars- (a) reforestation, (b) burnt, and (c) temperate.

**Figure 4:**
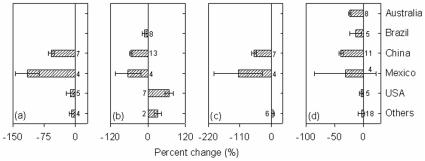
Percent change of ecosystem C pools in aboveground biomass (a) and litter mass (b), belowground biomass (c), and soil C stock (d) from natural forests to plantations in different geographic regions. Values near each bar indicated the number of cases synthesized.

The general pattern of the decreased ecosystem C pools in plantations relative to natural forests did not change between the two different groups in relation to various factors: stand age (<25 years vs. ≥25 years), stand types (broadleaved vs. coniferous and deciduous vs. evergreen), tree species origin (native vs. exotic) of plantations (Fig. 2), land-use history (afforestation vs. reforestation) and site preparation for plantations (unburnt vs. burnt treatment), and study regions (tropic vs. temperate) (Fig. 3). In addition, the pattern held true across geographic regions (Fig. 4).

## Discussion

The results obtained from this synthesis suggested some mechanisms underlying the difference in ecosystem C stock between plantations and natural forests. There were consistent decreases in ecosystem C pools with decreasing ANPP, aboveground litterfall and rate of soil respiration in plantations relative to natural forests. The decrease in fine root biomass could also explain the decreased amount of C input into plantations observed [21]. The decreases in soil available N, P and K concentrations were concerned with the lower litterfall in plantations relative to natural forests. In addition to the lower litterfall, the mean leaf litter N concentration, an important index of litter quality, was 14% lower in plantations than in natural forests, based upon 18 study cases from our literature. The lower soil nutrient availabilities in turn limit tree growth, and then constrain C sequestration in plantations. Thus, there was a potential negative feedback between ecosystem C cycle and plantations relative to natural forests.

### Methodological considerations

It is important to note that there were uncertainties in ecosystem C stock for this meta-analysis. There were not many formal field studies examining the difference in ecosystem C cycle between plantations and natural forests, as most data came from the studies that were not established specifically to address this issue. For some variables, the number of study cases were rather small (Table 1), and the weighted response ratio (*RR*_++_) might be sensitive to additions or deletions of published studies. Study sites were not randomly distributed in global forest ecosystems, and datasets compiled for this meta-analysis came from the regions where ecologists have extensively conducted relevant studies, while many other plantation regions have not attracted an attention from ecologists. These might cause biases in evaluation of the impacts of plantations. Thus, more experimental studies on ecosystem C cycle for plantations in comparison with natural forests are needed in the future. However, the general pattern of the decrease in ecosystem C pools in plantations relative to natural forests was independent of biomes, geographic regions or other factors (Figs. 2–4). The uncertainties are unlikely to change this general pattern.

### Altered ecosystem C processes

ANPP of plantations, with stand ages ranging from 32 to 50 years, was 11% lower than that of natural forests. The decrease in ANPP could result primarily from the differences in fine root biomass and leaf area index between plantations and natural forests. As well as fine root biomass (Fig. 1), leaf area index was significantly lower (−13%) in plantations than in natural forests based on nine field cases [23]–[26]. In addition, the reduction of ANPP could have resulted from decreased soil available N, P, and K concentrations in plantations relative to natural forests (Fig. 1). Due to the combined effects of reduced fine root biomass, leaf area index and soil nutrient availability, plantations might assimilate less atmospheric CO_2_ into ecosystems than natural forests. Our results on ANPP were inconsistent with the traditional opinion that plantations might have higher yield than natural forests [e.g., [5], [9], [14]]. The traditional opinion concerned the wood increment of main stems in plantations. All else being equal, the increment of stem wood is far smaller than ANPP in plantations.

Aboveground litterfall was 34% lower in plantations than in natural forests (Fig. 1), which is in agreement with many field studies [e.g., [6], [19]]. The decreased aboveground litterfall could be explained by the differences in ANPP and leaf area index between plantations and natural forests. Generally, a lower ANPP may generate less aboveground litter. The leaf portion may be high in aboveground litter stock [21], [27]. The lower leaf area index means that plantations had lower aboveground litterfall in comparison with natural forests. The decreased aboveground litterfall suggests that plantations might have decelerated ecosystem nutrient cycling processes in comparison with natural forests.

The rate of soil respiration was lower in plantations than in natural forests in 12 of 14 cases (Table 1). Belowground biomass and fine root biomass as well as soil microbial biomass are important to regulate the rate of soil respiration. Our results showed that all of belowground biomass, fine root biomass and soil microbial C concentration were lower in plantations than in natural forests. In addition, the change in soil respiration rate may be mediated by alteration of soil moisture [28]. A meta-analysis showed that soil moisture decreased by 25% in plantations relative to natural forests [29]. Interestingly, the rate of soil respiration might have been higher, otherwise soil C stock would not decrease in plantations when compared with natural forests. Of course, the reduction of soil C stock can also contribute to the decreased rate of soil respiration. Thus, it is necessary to quantify the overall change in other C fluxes and parameters of ecosystem, in addition to the rate of soil respiration, for a full understanding of the effects of plantations on soil C stock.

### Decreased ecosystem C stock

This meta-analysis demonstrated that ecosystem C pools, including those in above- and belowground biomass, aboveground litter mass and soil, was 28% lower in plantations than in natural forests. Our results about the amount of ecosystem C pools were consistent with those from studies by Dixon et al [4]. The decrease in ecosystem C stock is likely a combined result of both decreased NPP and litterfall, and the length of time since plantation establishment. On average, ANPP and aboveground litterfall decreased by 11% and 34% in plantations in comparison with natural forests, respectively. However, neither of *RR*s of the above two variables were observed to be significantly correlated with stand age of plantations (both *P*>0.1). The decreased ANPP leads to less atmospheric C, via photosynthesis, into plants and soils, meaning decreased soil C stock [21]. The decreased aboveground litterfall could result in less aboveground litter mass, and then less litter C incorporated into soils when the litter decomposed [27]. As a consequence, plantations sequestrated less C into ecosystems through the changes in ecosystem C fluxes.

Ecosystem C pools discussed above were statistically different between plantations and natural forests, such differences were affected by various factors (Figs. 2–4). High variabilities were observed in the differences between the two different groups in relation to these factors in our meta-analysis, indicating that caution is needed in predicting the differences on the basis of mean effects. Many of these factors are well known to affect ecosystem C pools [7], [8]. For example, stand age of plantations and site preparation for plantation establishment might have impact on the accumulation of aboveground biomass and litter, and then affect ecosystem C sequestration. In tropics, high mean annual precipitation and temperature might have stimulated tree growth, and thus more C is fixed into ecosystems [21]. *RR*s of soil C stock, for example, was not significantly correlated with stand age of plantations, latitude (north/south) and mean annual precipitation and temperature of the study sites (all *P*>0.1). Thus, the differences in ecosystem C pools between plantations and natural forests were related to the interactions of these factors. Any differential effects resulting from the two different groups in one of these factors could be swamped by the others for such differences.

### Implications

Our findings had at least two implications. First, plantations, with reduced ecosystem C stock, failed to function as C sink as originally intended, in comparison with natural forests. Over the last two decades, C sequestration strategies might have overstated the role of plantations in climate change mitigation [1], [8], [11], [30]. It is acknowledged that plantations established on non-forested fields such as agricultural lands do accumulate considerable C into woody biomass. However, a recent meta-analysis showed that conversion from non-forested lands to plantations caused a 6.7% decrease in soil C stock globally [22]. In addition, mean rate of soil uptake of CH_4_, another important greenhouse gas, significantly decreased by 80% in plantations when compared with the natural forests based on 11 field cases [31]–[35]. Moreover, on the lands where plantations can grow, if other conditions are equal, secondary forests can develop well through natural succession [e.g., [6], [12], [13]]. Thus, current strategies concerning C sequestration through creating plantations had better be adjusted by governments in international conferences like the United Nations' Climate Change Conference.

Second, our results on ecosystem C cycle provided an interpretation of ecosystem degradation associated with plantations [e.g., [6], [17], [36]]. For example, both plant biomass and soil organic C stock decreased respectively by 24 and 10% from the first to the second rotation for *C*. *lanceolata* plantations, and by 39% and 15% from second to the third rotation [37]. Of course, the decrease in ecosystem C stock was partially due to an increased output as plantations and (/or) wood products were harvested [3], [22]. Additionally, improperly silvicultural activities in plantations might have accelerated ecosystem C loss in plantations [6], [22], [36]. Site preparation with burnt treatment, for example, increased soil C loss, compared with unburnt one (Fig. 3b). To avoid ecosystem degradation associated with plantations, restoration measures need to be implemented to engineer ecosystems toward their natural potentials.

The shifts from natural forests to plantations can also generate other ecological problems. For example, soil bulk density, representing the degree of soil compaction, was 12.9% higher in plantations relative to natural forests [29]. Increased soil compaction may limit roots' access to water and nutrients, destroy soil structural units, slow gaseous diffusion, and reduce litter decomposition in plantations. Additionally, it has been reported that plantations decrease stream flow by 227 millimetres per year globally, and that climate feedbacks were unlikely to offset such water losses [38]. On the other hand, plantations can substantially provide human demands, e.g., domestic and industrial timbers. Therefore, we are now facing a great challenge of developing a management policy for plantation practice that minimizes their negative impacts on ecosystems but maximizes their traditional values.

## Materials and Methods

### Data sources

To avoid bias in publication selection, the following five criteria were set for the inclusion of data related to ecosystem C stock and other related variables for plantations and natural forests. First, the reference ecosystems relative to plantations were primary and secondary forests which were naturally generated and free from disturbance (i.e., natural forests). As a result, secondary forests were dominant in the reference ecosystems in this synthesis. Second, the trees in plantations were arboreal species, not including bamboos, shrubs, or fruit and non-timber species such as apple, coffee or rubber trees. Third, field studies were conducted by paired-site design in fields where there were both of plantations and natural forests [7], [8]. For studies conducted by chronosequence design for plantations compared with natural forests, the oldest plantations were included. For studies with repeated-sampling design for plantations compared with natural forests, the datasets sampled in the last time were collected. Fourth, studies were free of experimental treatments (e.g. free-air CO_2_ enrichment and warming) which did not belong to the normal range of silvicultural activities. Fifth, for soil variables, data were collected from the samples of soil surface layer. If data from the samples of different layers in a soil profile had been compiled into one, the compiled one was employed.

Databases of Blackwell, CNKI, Elsevier, Kluwer, JSTOR, Springer and Web of Science, licensed to Fudan University library, were used to search for source data from inception to September 2009. Study sites were located in all continents except for Antarctic. All the data used here were extracted from figures and tables in published papers. For each variable, the mean (M), standard error (SE) or deviation (SD) or 95%CI, and sample size (n) in both plantations and natural forests were extracted. Information on the factors such as stand age and types of plantations, land-use history and site preparation for plantations, and geographical conditions of study sites was collected. To examine the effects of these factors on ecosystem C pools, plantations were categorized into two different groups in relation to stand age (<25 years vs. ≥25 years), stand types (broadleaved vs. coniferous and deciduous vs. evergreen), tree species origin (native vs. exotic), land-use history (afforestation vs. reforestation) and site preparation for plantations (unburnt vs. burnt), and study regions (tropic vs. temperate). The threshold value of 25 years was determined by the common practice that mature plantation stands with fast growth rate are generally considered to be of less than 25 years in age. In addition, study sites were grouped into different geographic regions such as Australia, China and USA, and then the differences in ecosystem C pools between plantations and natural forests were examined in each of the geographic regions.

### Data analysis

The method of this meta-analysis followed previous studies [e.g., [39], [40]]. Plantations were regarded as treatment relative to natural forests. A response ratio (*RR*, the ratio of the mean value of a concerned variable in plantations to that in natural forests) was used here as an indicator of the difference in a variable between plantations and natural forests. To summarize the results from independent studies, weighted response ratio (*RR*_++_) was calculated from *RR*s to increase the precision of the combined estimate and the power of the tests. M, SE or SD or 95%CI, and *n* were used to compute *RR*, *RR*_++_ and 95%CI of *RR*_++_. Dixon's Q-test was performed to exclude outliers of *RR*s at α = 0.05. If the 95%CI value of *RR*_++_ for a variable did not overlap with zero, the variable was significantly different between plantations and natural forests. If the 95%CI value of *RR*_++_ for a variable did not overlap between the two different groups in relation to one of these factors: stand age, stand types and tree species origin of plantations, land-use history and site preparation for plantations, and study region, the *RR*_++_ was considered to be significantly different between the two groups and the factor has a significant effect on the variable. If the 95%CI value of *RR*_++_ overlapped, Student's-test was used to further examine the difference between the two different groups, which was considered to be significant at the level of *P*<0.05. The percent change in a variable from natural forests to plantations was calculated by [exp (*RR*_++_)−1] ×100%.

A simple model: *Y* = *a* [1-exp (−b/x)], was used to calculate the mean soil C stock within the depth of 100 cm in both plantations and natural forests, where *x* was the depth of sampled soil, *Y* was soil C stock, *a* and *b* were estimated parameters. For the regressions fitted here, correlation coefficient (*R*) was larger than 0.52, and statistical *P* value was less than 0.001 for both plantations and natural forests. Soil C stocks within a depth of 100 cm and their standard errors were derived from the fitted equations.

## Supporting Information

Table S1 RR (N = 1) or RR++ (N>1) and the number of cases (N, in parentheses) for thirteen variables extracted from each of the 86 papers (Reference list follows in supplementary references S1).
(0.26 MB DOC)

References S1 List of 86 papers from which datasets of the thirteen variables were extracted for this meta-analysis.
(0.10 MB DOC)
