# Supplementary material for: Ecosystem Carbon Stock Influenced by Plantation Practice: Implications for Planting Forests as a Measure of Climate Change Mitigation
Source: PLoS One. 2010 May 27;5(5):e10867. doi: 10.1371/journal.pone.0010867 (PMC2877715; doi:10.1371/journal.pone.0010867)
Supplement: Table S1 — RR (N = 1) or RR++ (N>1) and the number of cases (N, in parentheses) for thirteen variables extracted from each of the 86 papers (Reference list follows in supplementary references S1). (0.26 MB DOC) [file pone.0010867.s001.doc]

Table S1— *RR* (*N* = 1) or *RR*_++_ (*N* > 1) and the number of cases (*N*, in parentheses) for thirteen variables extracted from each of the 86 papers (Reference list follows in supplementary references S1).

| No. | Citation | (1) | (2) | (3) | (4) | (5) | (6) | (7) | (8) | (9) | (10) | (11) | (12) | (13) |
| --- | --- | --- | --- | --- | --- | --- | --- | --- | --- | --- | --- | --- | --- | --- |
| 1 | Aborisade & Aweto,1990 |  |  |  |  |  |  |  |  | -0.20  (2) |  |  | -0.09  (2) |  |
| 2 | An et al., 2007 |  |  |  |  |  |  |  | -1.22  (1) | -0.08  (1) |  |  |  |  |
| 3 | Arevalo et al., 2009 |  |  |  | -0.22  (1) |  | -0.87  (1) | 0.35  (1) | -0.20  (1) |  | 0.58  (1) |  |  |  |
| 4 | Ashagrie et al., 2005 |  |  |  |  |  |  | -0.11  (1) |  | -0.17  (1) |  |  |  |  |
| 5 | Attignon et al., 2004 |  |  |  |  |  |  |  |  | -0.49  (2) |  |  |  |  |
| 6 | Aweto & Moleele 2005 |  |  |  |  |  |  |  |  | 0.00  (1) |  |  | 0.08  (1) |  |
| 7 | Barlow et al., 2007 |  | -0.54  (2) |  |  |  |  |  |  |  |  |  |  |  |
| 8 | Başaran et al., 2008 |  |  |  |  |  |  |  |  | 0.05  (1) |  |  |  |  |
| 9 | Bayramin et al., 2007 |  |  |  |  |  |  |  |  | 0.05  (1) |  |  |  |  |
| 10 | Behera & Sahani 2003 |  |  |  |  |  |  |  |  | -0.33  (2) | -1.06  (2) |  |  |  |
| 11 | Bernhard-Reversat 1988 |  |  |  |  |  |  |  |  | -0.72  (1) |  |  |  |  |
| 12 | Cassagne et al., 2004 |  |  |  |  |  |  |  |  | -0.15  (1) |  |  |  |  |
| 13 | Cavelier & Tobler 1998 |  |  |  |  |  |  |  |  | -0.32  (2) |  |  |  |  |
| 14 | Chen et al., 2005 |  | -0.39  (4) |  | -0.69  (4) | -0.69  (4) | -0.82  (4) | -0.35  (4) | -0.90  (4) | -0.45  (4) |  | -0.15  (4) | -0.29  (4) | -0.25  (4) |
| 15 | Chen & Li 2003 |  |  |  |  |  |  |  |  | -1.35  (1) |  |  |  |  |
| 16 | Cromack Jr et al., 1999 |  |  |  | -1.75  (1) |  |  |  |  |  |  |  |  |  |
| 17 | Cuevas et al., 1991 |  | 0.39  (1) |  | 0.98  (1) | 0.74  (1) |  | -0.02  (1) | -2.35  (1) | -0.19  (1) |  |  |  |  |
| 18 | Deng & Shangguan 2009 |  |  |  |  |  |  |  |  | 0.19  (2) |  |  |  |  |
| 19 | Fang et al., 2006 |  |  |  |  |  |  |  |  | -0.49  (2) |  |  |  |  |
| 20 | Firn et al., 2007 |  |  |  |  |  |  |  |  |  |  | -0.20  (6) | -0.27  (6) |  |
| 21 | Goma-Tchimbakala &  Bernhard-Reversat 2006 |  |  |  |  | 0.32  (1) |  |  |  |  |  |  |  |  |
| 22 | Gong & Liao 2009 |  |  |  |  |  |  |  |  | -0.27  (1) |  |  |  |  |
| 23 | Gong et al., 2007 |  |  |  |  |  |  |  |  | -0.70  (2) |  |  |  |  |
| 24 | He et al., 2006 |  |  |  |  |  |  |  |  |  | -0.77  (1) |  |  | -0.21  (1) |
| 25 | Holt & Spain 1986 |  |  |  |  |  |  |  |  | -0.13  (1) |  |  |  |  |
| 26 | Hu et al., 2005 |  |  | -0.77  (3) |  |  |  |  |  | -0.57  (3) |  |  |  |  |
| 27 | Inagaki et al., 2004 |  |  |  |  |  |  |  |  | 0.02  (4) |  | 0.49  (4) |  |  |
| 28 | Ishizuka et al., 2005 |  |  | 0.03  (1) |  |  |  |  |  |  |  |  |  |  |
| 29 | Kasel & Bennett 2007 |  |  |  |  |  |  | -0.18  (4) |  | -0.34  (4) | -0.15  (4) |  |  |  |
| 30 | Kilpeläinen et al., 2007 |  |  |  |  |  |  | 0.38  (1) |  | 0.31  (1) |  |  | 0.00  (1) |  |
| 31 | Kranabetter & Macadam 2007 |  |  |  |  |  |  | -0.37  (1) |  | -0.39  (1) |  |  |  |  |
| 32 | Laclau 2003 |  |  |  | -0.42  (1) | -0.25  (1) | 0.28  (1) | -0.32  (1) | 0.68  (1) |  |  |  |  |  |
| 33 | Lemenih et al., 2004 |  |  |  |  |  |  |  |  | -0.50  (2) |  |  | 0.04  (2) | 0.84  (2) |
| 34 | Lemma et al., 2006 |  |  |  |  |  |  | -0.05  (3) |  | -0.49  (3) |  |  |  |  |
| 35 | Li et al., 2005 |  | 0.20  (1) | -0.13  (1) |  | 0.99  (1) |  | -0.05  (1) |  |  | -0.18  (1) |  |  |  |
| 36 | Li et al., 2004 |  |  |  |  | -1.78  (1) |  | -0.56  (1) |  |  |  |  |  |  |
| 37 | Lin et al., 2006 |  | -0.05  (2) |  |  | -1.03  (2) |  |  |  |  |  |  |  |  |
| 38 | Livesley et al., 2009 |  |  | -0.27  (2) |  |  |  |  |  |  |  |  |  |  |
| 39 | Lugo 1992 |  |  |  |  | 0.74  (4) |  |  | -1.22  (4) |  |  |  |  |  |
| 40 | Liu et al., 2002 |  |  |  |  |  |  |  |  | -0.32  (2) |  | -0.09  (2) | 0.00  (2) | -0.54  (2) |
| 41 | Macedo et al., 2008 |  |  |  |  |  |  | -0.20  (1) |  | 0.06  (1) |  |  |  |  |
| 42 | Marcos et al., 2007 |  |  |  |  |  |  |  |  | -0.30  (1) |  |  |  |  |
| 43 | Markewitz et al., 2002 |  |  |  |  |  |  | -0.88  (1) |  | -1.07  (1) |  |  |  |  |
| 44 | Martius et al., 2004 |  | -0.14  (4) |  |  | -0.22  (4) |  |  |  |  |  |  |  |  |
| 45 | McClaugherty et al., 1982 |  |  |  |  | -0.41  (1) |  |  | -0.13  (1) |  |  |  |  |  |
| 46 | Mo et al., 2009 |  |  |  |  | 0.62  (1) |  |  |  |  |  |  |  |  |
| 47 | Mo et al., 2006 |  |  |  |  |  |  |  |  | -0.44  (1) |  |  |  |  |
| 48 | Morris et al., 2007 |  |  |  |  |  |  | -0.06  (2) |  |  |  |  |  |  |
| 49 | Nihlgård 1972 | -0.12  (1) | 0.00  (1) |  | -0.05  (1) |  | 0.15  (1) |  |  |  |  |  |  |  |
| 50 | Ordόñez et al., 2008 |  |  |  | -0.77  (4) | -0.49  (4) | -0.77  (4) |  |  |  |  |  |  |  |
| 51 | Oseni et al., 2007 |  |  |  |  |  |  |  |  | 0.83  (1) | -2.50  (1) |  |  |  |
| 52 | Pangle et al., 2009 | -0.15  (3) |  |  | -0.08  (3) |  |  |  |  |  |  |  |  |  |
| 53 | Parfitt et al., 2003 |  |  |  |  |  |  | -0.03  (1) |  |  |  |  |  |  |
| 54 | Pibumrung et al., 2008 |  |  |  | -1.51  (1) |  |  | -0.12  (1) | -0.32  (1) |  |  |  |  |  |
| 55 | Pinzari et al., 1999 |  |  | -0.67  (3) |  |  |  |  |  | -0.13  (3) | 0.03  (3) |  |  |  |
| 56 | Reich et al., 1997 | -0.01  (3) |  |  |  |  |  |  |  |  |  |  |  |  |
| 57 | Richards et al., 2007 |  |  |  |  |  |  | -0.25  (4) |  |  |  |  |  |  |
| 58 | Russell et al., 2007 |  |  |  |  |  |  | -0.14  (6) |  |  |  |  |  |  |
| 59 | Santa Regina & Tarazona 2000 |  | 0.21  (1) |  |  |  |  |  |  |  |  |  |  |  |
| 60 | Schmitz et al., 1998 |  |  |  |  |  |  |  |  | 0.00  (1) |  |  |  |  |
| 61 | Smith et al., 2002 |  | -0.02  (4) |  |  | 0.21  (4) |  | -0.12  (4) | -0.08  (4) |  |  |  |  |  |
| 62 | Smith et al., 1998 |  |  |  |  |  |  |  |  | -0.05  (4) |  |  | 0.05  (4) |  |
| 63 | Solomon et al., 2002 |  |  |  |  |  |  |  |  | -0.37  (1) |  |  |  |  |
| 64 | Tamooh et al., 2008 |  |  |  |  |  | 0.26  (3) |  |  |  |  |  |  |  |
| 65 | Tang et al., 2006 |  |  | -0.18  (1) |  |  |  |  | -0.56  (1) |  |  |  |  |  |
| 66 | Tateno et al., 2007 |  | -0.10  (1) |  |  |  |  | 0.08  (1) |  | -0.49  (1) |  |  |  |  |
| 67 | Wall & Hytönen 2005 |  |  |  |  |  |  | 0.32  (2) |  | 0.34  (2) |  |  |  |  |
| 68 | Wang et al., 2007 |  |  |  |  |  |  | -1.04  (2) |  | -1.09  (2) |  |  |  |  |
| 69 | Wang et al., 2009 |  |  |  |  |  |  |  |  | -0.20  (4) |  | -0.77  (4) | 0.02  (4) | -1.14  (4) |
| 70 | Wang et al., 2008 |  |  |  |  |  |  |  |  | -0.52  (1) |  |  |  |  |
| 71 | Wang & Wang 2007 |  |  |  |  |  |  |  |  | -0.18  (1) | -0.34  (1) |  |  |  |
| 72 | Wang et al., 2005 |  |  |  |  |  |  |  |  | -0.46  (1) | -0.80  (1) |  | 0.03  (1) | -0.21  (1) |
| 73 | Wang et al., 2006 |  |  |  |  | -0.07  (2) |  |  |  | -0.78  (1) | -0.75  (1) |  |  |  |
| 74 | Xu & Liu 2004 |  |  |  |  |  |  |  |  | -1.52  (1) |  | -1.54  (1) | -1.02  (1) | -0.59  (1) |
| 75 | Yamashita et al, 2008 |  |  |  |  |  |  |  |  | -0.11  (1) |  |  |  | 0.18  (1) |
| 76 | Yang & Xie 2002 |  |  |  |  |  |  |  |  |  |  | 0.05  (1) | 0.13  (1) | 0.14  (1) |
| 77 | Yang et al., 2007 | -0.02  (2) |  | -0.72  (2) |  |  |  |  |  |  |  |  |  |  |
| 78 | Yang et al., 2005 (a) |  |  |  | -0.33  (1) | -0.69  (1) | -0.59  (1) | -0.09  (1) | -0.69  (1) | -0.29  (1) |  |  |  |  |
| 79 | Yang et al., 2005(b) |  | -0.43  (7) |  |  |  |  |  |  |  |  |  |  |  |
| 80 | Zeller et al., 2007 |  |  |  |  |  |  |  |  | 0.15  (1) |  |  |  |  |
| 81 | Zhang et al., 2004 (a) |  |  |  |  |  |  |  |  |  | -0.10  (1) |  |  |  |
| 82 | Zhang et al., 2004 (b) |  |  |  |  |  |  |  |  | -0.93  (1) |  | 0.01  (1) | -0.33  (1) | 0.36  (1) |
| 83 | Zheng et al., 2008 |  |  |  | -0.44  (2) | -0.42  (2) | -0.33  (2) | -0.26  (2) |  |  |  |  |  |  |
| 84 | Zheng et al., 2004 |  |  |  |  |  |  |  |  | -0.43(2) | -0.37  (2) | -0.53  (2) | -0.18  (2) | -0.90  (2) |
| 85 | Zhou et al., 2007 |  |  | -0.20  (1) |  |  |  |  |  |  |  |  |  |  |

Note: (1) aboveground net primary production, (2) rate of litterfall, (3) rate of soil respiration, (4) aboveground biomass, (5) aboveground litter mass, (6) belowground biomass, (7) soil C stock, (8) fine root biomass, (9) soil C concentration (%), (10) soil microbial C concentration (%), (11) available soil N, (12) P and (13) K concentrations (%).
