# Supplementary material for: Ecosystem Carbon Stock Influenced by Plantation Practice: Implications for Planting Forests as a Measure of Climate Change Mitigation
Source: PLoS One. 2010 May 27;5(5):e10867. doi: 10.1371/journal.pone.0010867 (PMC2877715; doi:10.1371/journal.pone.0010867)
Supplement: References S1 — List of 86 papers from which datasets of the thirteen variables were extracted for this meta-analysis. (0.10 MB DOC) [file pone.0010867.s002.doc]

References S1— List of 86 papers from which datasets of the thirteen variables were extracted for this meta-analysis.

1. Aborisade KD, Aweto AO (1990) Effects of exotic tree plantations of teak (*Tectona grandis*) and gmelina (*Gmelina arborea*) on a forest soil in south-western Nigeria. Soil Use and Management 6:43-45.
2. An H, Wei LY, Liu Y, Shangguan ZP (2007) Distribution characters of fine root of artificial *Pinus* *tabulaeformis* and natural *Betula platyphylla* forests and their relation to soil nutrients in Hilly Loess regions (*In Chinese*). Plant Nutrition and Fertilizer Science 13:611-619.
3. Arevalo CBM, Bhatti[b](http://www.sciencedirect.com/science?_ob=ArticleURL&_udi=B6T6X-4VP1CMS-4&_user=10&_rdoc=1&_fmt=&_orig=search&_sort=d&view=c&_acct=C000050221&_version=1&_urlVersion=0&_userid=10&md5=d4151389e6150df87d7e2712bad10bf3#aff2%23aff2) JS, Chang SX, Sidders D (2009) Ecosystem carbon stocks and distribution under different land-uses in north central Alberta, Canada. Forest Ecology and Management 257:1776-1785.
4. Ashagrie Y, Zech W, Guggenberger G (2005) Transformation of a *Podocarpus falcatus* dominated natural forest into a monoculture *Eucalyptus globulus* plantation at Munesa, Ethiopia: soil organic C, N and S dynamics in primary particle and aggregate-size fractions. Agriculture, Ecosystems and Environment 106:89-98.
5. Attignon SE, Weibel D, Lachat T, Sinsin B, Nagel P, et al. (2004) Leaf litter breakdown in natural and plantation forests of the Lama forest reserve in Benin. Applied Soil Ecology 27:109-124.
6. Aweto AO, Moleele NM (2005) Impact of *Eucalyptus camaldulensis* plantation on an alluvial soil in south eastern Botswana. International Journal of Environmental Studies 62:163-170.
7. Barlow J, Gardner TA, Ferreira LV, Peres CA (2007) Litter fall and decomposition in primary, secondary and plantation forests in the Brazilian Amazon. Forest Ecology and Management 247:91-97.
8. Başaran M, Erpul G, Tercan AE, Çanga MR (2008) The effects of land use changes on some soil properties in İndaği Mountain Pass-Çankiri, Turkey. Environmental Monitoring and Assessment 136:101-119.
9. Bayramin İ, Basaran M, Erpul G, Canga MR (2007) Assessing the effects of land use changes on soil sensitivity to erosion in a highland ecosystem of semi-arid Turkey. Environmental Monitoring and Assessment 140:249-265.
10. Behera N, Sahani U (2003) Soil microbial biomass and activity in response to *Eucalyptus* plantation and natural regeneration on tropical soil. Forest Ecology and Management 174:1-11.
11. Bernhard-Reversat F (1988) Soil nitrogen mineralization under a *Eucalyptus* plantation and a natural Acacia forest in Senegal. Forest Ecology and Management 23:233-244.
12. Cassagne N, Bal-Serin MC, Gers C, Gauquelin T (2004) Changes in humus properties and collembolan communities following the replanting of beech forests with spurce. Pedobiologia 48:267-276.
13. Cavelier J, Tobler A (1998) The effect of abandoned plantations of *Pinus patula* and *Cupressus lusitanica* on soils and regeneration of a tropical montane rain forest in Colombia. Biodiversity and Conservation 7:335-347.
14. Chen GS, Yang YS, Xie JS, Guo JF, Gao R, et al. (2005) Conversion of a natural broad-leafed evergreen forest into pure plantation forests in a subtropical area: effects on carbon storage. Annual Forestry Science 62:659-668.
15. Chen XW, Li BL (2003) Change in soil carbon and nutrient storage after human disturbance of a primary Korean pine forest in Northeast China. Forest Ecology and Management 186:197-206.
16. Cromack Jr K, Miller RE, Helgerson OT, Smith RB, Anderson HW (1999) Soil carbon and nutrients in a coastal Oregon Douglas-fir plantation with red alder. Soil Society of America Journal 63:232-239.
17. Cuevas E, Brown S, Lugo AE (1991) Above- and belowground organic matter storage and production in a tropical pine plantation and a paired broadleaf secondary forest. Plant and soil 135:257-268.
18. Deng J, Shangguan ZP (2009) Nutrient and carbon pools in both natural and artificial *Pinus tabulaeformis* in Ziwuling region (*In Chinese*). Acta Ecologica Sinica 29:3231-3240.
19. Fang YT, Zhu WX, Mo JM, Zhou GY, Gundersen P (2006) Dynamics of soil inorganic nitrogen and their responses to nitrogen additions in three subtropical forests, south China. Journal of Environmental Science 18:752-759.
20. Firn J, Erskine PD, Lamb D (2007) Woody species diversity influences productivity and soil nutrient availability in tropical plantations. Oecologia 154:521-533.
21. Goma-Tchimbakala J, Bernhard-Reversat F (2006) Comparison of litter dynamics in three plantations of an indigenous timber-tree species (*Terninalia superba*) and a natural tropical forest in Mayombe, Congo. Forest Ecology and Management 229:304-313.
22. Gong SS, Liao SG (2009) Soil nutrient characteristics in eucalypt plantation and natural forest (*in Chinese*). Journal of Jiangsu Forestry Science & Technology 36:1-4.
23. Gong W, Hu TX, Wang JY, Gong YB, Ran H (2007) Impacts of litter on soil in the natural evergreen broadleaved forests after artificial regeneration in southern Sichuan (*In Chinese*). Scientia Silvae Sinicae 43:112-119.
24. He YJ, Wang QK, Wang SL, Yu XJ (2006) Characteristics of soil microbial biomass carbon and nitrogen and their relationships with soil nutrients in *Cunninghamia lanceolata* plantations (*In Chinese*). Chinese Journal of Applied Ecology 17:2292-2296.
25. Holt JA, Spain AV (1986) Some biological and chemical changes in a North Queensland soil following replacement of rainforest with *Araucaria cunninghamii* (Coniferae: Araucariaceae). The Journal of Applied Ecology 23:227-237.
26. Hu YL, Wang SL, Yan SK, Gao H (2005) Effects of replacing natural secondary broad-leaved forest with *Cunninghamia lanceolata* plantation on soil biological activities (*In Chinese*). Chinese Journal of Applied Ecology 16:1411-1416.
27. Inagaki Y, Miura S, Kohzu A (2004) Effects of forest type and stand age on litterfall quality and soil N dynamics in Shikoku district, southern Japan. Forest Ecology and Management 202:107-117.
28. Ishizuka S, Iswandi A, Nakajima Y, Yonemura S, Sudo S, et al. (2005) The variation of greenhouse gas emissions from soils of various land-use cover types in Jambi province, Indonesia. Nutrient Cycling in Agroecosystems 71:17-32.
29. Kasel S, Bennett LT (2007) Land-use history, forest conversion, and soil organic carbon in pine plantations and native forests of south eastern Australia. Geoderma 137:401-413.
30. Kilpeläinen J, Finér L, Niemelä P, Domisch T, Neuvonen S, et al. (2007) Carbon, nitrogen and phosphorus dynamics of ant mounds (*Formica rufa* group) in managed boreal forests of different successional stages. Applied Soil Ecology 36:156-163.
31. Kranabetter JM, Macadam AM (2007) Changes in carbon storage of broadcast burn plantations over 20 years. Canadian Journal of Soil Science 87:93-102.
32. Laclau P (2003) Biomass and carbon sequestration of ponderosa pine plantations and native cypress forests in northwest Patagonia. Forest Ecology and Management 180:317-333.
33. Lemenih M, Olsson M, Karltun E (2004) Comparison of soil attributes under *Cupressus lusitanica* and *Eucalyptus saligna* established on abandoned farmlands with continuously cropped farmlands and natural forest in Ethiopia. Forest Ecology and Management 195:57-67.
34. Lemma B, Kleja DB, Nilsson I, Olsson M (2006) Soil carbon sequestration under different exotic tree species in the southwestern highlands of Ethiopia. Geoderma 136:886-898.
35. Li YQ, Xu M, Zou XM, Shi PJ, Zhang YQ (2005) Comparing soil organic carbon dynamics in plantation and secondary forest in wet tropics in Puerto Rico. Global Change Biology 11:239-248.
36. Li ZC, Fu MY, Xie JZ, Zhou BZ, Xiao TQ, et al. (2004) Carbon sequestration of 5 ecological reestablishment vegetation types in Muchuan County of Sichuan (*In Chinese*). Journal of Zhejiang Forestry College 21:382-387.
37. Lin B, Liu Q, Wu Y, He H (2006) Nutrient and litter patterns in three subalpine coniferous forests of western Sichuan, China. Pedosphere 16:380-389.
38. Liu SL, Fu BJ, Lü YH, Chen LD (2002) Effects of reforestation and deforestation on soil properties in humid mountainous areas a case study in Wolong Nature Reserve, Sichuan province, China. Soil Use and Management 18:376-380.
39. Livesley SJ, Kiese R, Miehle P, Weston CJ, Butterbach-Bahl K, et al. (2009) Soil-atmosphere exchange of greenhouse gases in a *Eucalyptus marginata* woodland, a clover-grass pasture, and *Pinus radiata* and *Eucalyptus globulus* plantations. Global Change Biology 15:425-440.
40. Lugo AE (1992) Comparison of tropical tree plantations with secondary forests of similar age. Ecological Monographs 62:1-41.
41. Macedo MO, Resende AS, Garcia PC, Boddey RM, Jantalia CP, et al. (2008) Changes in soil C and N stocks and nutrient dynamics 13 years after recovery of degraded land using leguminous nitrogen-fixing trees. Forest Ecology and Management 255:1516-1524.
42. Marcos JA, Marcos E, Taboada A, Tárrega R (2007) Comparison of community structure and soil characteristics in different aged *Pinus sylvestris* plantations and a natural pine forest. Forest Ecology and Management 247:35-42.
43. Markewitz D, Sartori F, Craft C (2002) Soil change and carbon storage in longleaf pine stands planted on marginal agricultural lands. Ecological Applications 12:1276-1285.
44. Martius C, Höfer H, Garcia MVB, Römbke J, Hanagarth W (2004) Litter fall, litter stocks and decomposition rates in rainforest and agroforestry sites in central Amazonia. Nutrient Cycling in Agroecosystems 68:137-154.
45. McClaugherty CA, Aber JD, Melillo JM (1982) The role of fine roots in the organic matter and nitrogen budgets of two forested ecosystems. Ecology 63:1481-1490.
46. Mo F, Yu PT, Wang YH, Wang J, Wei X, et al. (2009) The water-holding capacity of litter layers in the forests of *Larix principisrupprechtii* and *Betula albo-sinesis* in Liupan Mountain and their rainfall interception process (*In Chinese*). Acta Ecologica Sinica 29:2868-2876.
47. Mo JM, Fang YT, Lin ED, Li YE (2006) Soil N_2_O emission and its response to simulated N deposition in the main forests of Dinghushan in subtropical China (*In Chinese*). Journal of Plant Ecology 30:901-910.
48. Morris SJ, Bohm S, Haile-Mariam S, Paul EA (2007) Evaluation of carbon accrual in afforested agricultural soils. Global Change Biology 13:1145-1156.
49. Nihlgård B (1972) Plant biomass, primary production and distribution of chemical elements in a beech and a planted spruce forest in South Sweden. Oikos 23:69-81.
50. Ordόñez JAB, de Jong BHJ, garcía-Oliva F, Aviña FL, Pérez JV, et al. (2008) Carbon content in vegetation, litter, and soil under 10 different land-use and land-cover classes in the Central Highlands of Michoacan, Mexico. Forest Ecology and Management 255:2074-2084.
51. Oseni OA, Ekperigin MM, Akindahunsi AA, Oboh G (2007) Studies of physiochemical and microbial properties of soils from rainforest and plantation in Ondo state Nigeria. African Journal of Agricultural Research 2:605-609.
52. Pangle K, Vose JM, Teskey RO (2009) Radiation use efficiency in adjacent hardwood and pine forests in the southern Appalachians. Forest Ecology and Management 257:1034-1042.
53. Parfitt RL, Scott NA, Ross DJ, Salt GJ, Tate KR (2003) Land-use change effects on soil C and N transformations in soils of high N status: comparisons under indigenous forest, pasture and pine plantation. Biogeochemistry 66:203-221.
54. Pibumrung P, Gajaseni N, Popan A (2008) Profiles of carbon stocks in forests, reforestation and agricultural land, Northern Thailand. Journal of Forestry Research 19:11-18 (DOI:10.1007/s11676-008-0002-y).
55. Pinzari F, Trinchera A, Benedetti A, Sequi P (1999) Use of biochemical indices in the mediterranean environment comparison among soils under different forest vegetation. Journal of Microbiological Methods 36:21-28.
56. Reich PB, Grigal DF, Aber JD, Gower ST (1997) Nitrogen mineralization and productivity in 50 hardwood and conifer stands on diverse soils. Ecology 78:335-347.
57. Richards AE, Dalal RC, Schmidt S (2007) Soil carbon turnover and sequestration in native subtropical tree plantations. Soil Biology & Biochemistry 39:2078-2090.
58. Russell AE, Raich JW, Valverde-Barrantes OJ, Fisher RF (2007) Tree species effects on soil properties in experimental plantations in tropical moist forest. Soil Science Society of America Journal 71:1389-1397.
59. Santa Regina I, Tarazona I (2000) Nutrient return to the soil through litterfall and throughfall under beech and pine stands of Sierra de la Demanda, Spain. Arid Soil Research and Rehabilitation, 14:239-252.
60. Schmitz MF, Atauri JA, de Pablo CL, de Agar PM, Rescia AJ, et al. (1998) Changes in land use in Northern Spain: Effects of forestry management on soil conservation. Forest Ecology and Management 109:137-150.
61. Smith CF, de Assis Oliveira F, Gholz HL, Baima A (2002) Soil carbon stocks after forest conversion to tree plantations in lowland Amazonia, Brazil. Forest Ecology and Management 164:257-263.
62. Smith CF, Gholz HL, de Assis Oliveira F (1998) Soil nitrogen dynamics and plant-induced soil changes under plantations and primary forest in lowland Amazonia, Brazil. Plant and Soil 200:193-204.
63. Solomon D, Lehmann J, Mamo T, Fritzsche F, Zech W (2002) Phosphorus forms and dynamics as influenced by land use changes in sub-humid Ethiopian highlands. Geoderma 105:21-48.
64. Tamooh F, Huxham M, Karachi M, Mencuccini M, Kairo JG, et al. (2008) Below-ground root yield and distribution in natural and replanted mangrove forests at Gazi bay, Kenya. Forest Ecology and Management 256:1290-1297.
65. Tang XL, Liu SG, Zhou GY, Zhang DQ, Zhou CY (2006) Soil-atmospheric exchange of CO_2_, CH_4_, and N_2_O in three subtropical forest ecosystems in southern China. Global Chang Biology 12:546-560.
66. Tateno R, Tokuchi N, Yamanaka N, Du S, Otsuki K, et al. (2007) Comparison of litterfall production and leaf litter decomposition between an exotic black locust plantation and an indigenous oak forest near Yan’an on the Loess Plateau, China. Forest Ecology and Management 241:84-90.
67. Wall A, Hytönen J (2005) Soil fertility of afforested arable land compared to continuously forested sites. Plant and Soil 275:247-260.
68. Wang CM, Ouyang H, Shao B, Tian YQ, Zhao JG, et al. (2006) Soil carbon changes following afforestation with Olga Bay Larch (*Larix olgensis* Henry) in Northeastern China. Journal of Integrative Plant Biology 48:503-512.
69. Wang CM, Liu YH, Shao B, Zhao JG (2007) Quantifying the soil carbon changes following the afforestation of former arable land (*In Chinese*). Journal of Beijing Forestry University 29:112-119.
70. Wang HY, Lei XD, Lu YC, Zhou YH, He CL (2009) Comparisons of soil chemical properties under four typical forest stands in Hainan province (*In Chinese*). Forest Research 22:129-133.
71. Wang L, Wang QJ, Wei SP, Shao MA, Li Y (2008) Soil desiccation for Loess soils on natural and regrown areas. Forest Ecology and Management 255:2467-2377.
72. Wang QK, Wang SL (2007) Soil organic matter under different forest types in Southern China. Geoderma 142:349-356.
73. Wang QK, Wang SL, Deng SJ (2005) Comparative study on active soil organic matter in Chinese fir plantation and native broad-leaved forest in subtropical China. Journal of Forestry Research 16:23-26.
74. Wang QK, Wang SL, Feng ZW (2006) Comparison of active soil organic carbon pool between Chinese fir plantations and evergreen broadleaved forests (*In Chinese*). Journal of Beijing Forestry University 28:1-6.
75. Xu MX, Liu GB (2004) The characteristics and evolution of soil nutrient in artificial black locust (*Robinia pseudoascacia*) forest land in the hilly Loess Plateau (*In Chinese*). Plant Nutrition and Fertilizer Science 10:40-46.
76. Yamashita N, Ohta S, Hardjono A (2008) Soil changes induced by *Acacia mangium* plantation establishment: Comparison with secondary forest and *Imperata cylindrica* grassland soil in South Sumatra, Indonesia. Forest Ecology and Management 254:362-370.
77. Yang MS, Xie HC (2002) Effects of introduced larch forests on soil (*In Chinese*). Journal of Northwest Forestry University 17:35-37.
78. Yang YS, Chen GS, Guo JF, Xie JS, Wang XG (2007) Soil respiration and carbon balance in a substropical native forest and two managed plantations. Plant Ecology 193:71-84.
79. Yang YS, Guo JF, Chen GS, Xie JS, Gao R, et al. (2005a) Carbon and nitrogen pools in Chinese fir and evergreen broadleaved forests and changes associated with felling and burning in mid-subtropical China. Forest Ecology and Management 216:216-226.
80. Yang YS, Guo JF, Chen GS, Xie JS, Gao R, et al. (2005b) Litter production, seasonal pattern and nutrient return in seven natural forests compared with a plantation in southern China. Forestry 78:403-415.
81. Zeller B, Brechet C, Maurice J-P, Le Tacon F (2007) ^13^C and ^15^N isotopic fractionation in trees, soils and fungi in a natural forest stand and a Norway spruce plantation. Annals of Forest Science 64:419-429.
82. Zhang WJ, Feng JX, Wu J, Parker K (2004a) Differences in soil microbial biomass and activity for six agroecosystems with a management disturbance gradient. Pedosphere 14:441-447.
83. Zhang YM, Zhou GY, Wu N, Bao WK (2004b) Soil enzyme activity changes in different-aged spruce forests of the eastern Qinghai-Tibetan Plateau. Pedosphere 14:305-312.
84. Zheng H, Ouyang ZY, Wang XK, Hong M, Zhao TQ, et al. (2004) Effects of forest restoration types on soil quality in red soil eroded region, Southern China (*In Chinese*). Acta Ecologica Sinica 24:1994-2002.
85. Zheng H, Ouyang ZY, Xu WH, Wang XK, Miao H, et al. (2008) Variation of carbon storage by different reforestation types in the hilly red soil region of southern China. Forest Ecology and Management 255:1113-1121.
86. Zhou HX, Zhang YD, Sun HL, Wu SY (2007) Soil respiration in temperate secondary forest and *Larix gmelinii* plantation in northeast China (*In Chinese*). Chinese Journal of Applied Ecology 18:2668-2674.
